# Telomeres shorten and then lengthen before fledging in Magellanic penguins (*Spheniscus magellanicus*)

**DOI:** 10.18632/aging.101172

**Published:** 2017-02-08

**Authors:** Jack A. Cerchiara, Rosa Ana Risques, Donna Prunkard, Jeffrey R. Smith, Olivia J. Kane, P. Dee Boersma

**Affiliations:** ^1^ Department of Biology, University of Washington, Seattle, WA 98195, USA; ^2^ Department of Pathology, University of Washington, Seattle, WA 98195, USA; ^3^ School of Environmental and Forest Sciences, University of Washington, Seattle, WA 98195, USA; ^4^ Wildlife Conservation Society, Bronx, NY 10460, USA; ^5^ Global Penguin Society, University of Washington, Seattle, WA 98195, USA

**Keywords:** telomeres, growth, life history, aging

## Abstract

For all species, finite metabolic resources must be allocated toward three competing systems: maintenance, reproduction, and growth. Telomeres, the nucleoprotein tips of chromosomes, which shorten with age in most species, are correlated with increased survival. Chick growth is energetically costly and is associated with telomere shortening in most species. To assess the change in telomeres in penguin chicks, we quantified change in telomere length of wild known-age Magellanic penguin (*Spheniscus magellanicus*) chicks every 15 days during the species’ growth period, from hatching to 60 days-of-age. Magellanic penguins continue to grow after fledging so we also sampled a set of 1-year-old juvenile penguins, and adults aged 5 years. Telomeres were significantly shorter on day 15 than on hatch day but returned to their initial length by 30 days old and remained at that length through 60 days of age. The length of telomeres of newly hatched chicks, chicks aged 30, 45 and 60 days, juveniles, and adults aged 5 years were similar. Chicks that fledged and those that died had similar telomere lengths. We show that while telomeres shorten during growth, Magellanic penguins elongate telomeres to their length at hatch, which may increase adult life span and reproductive opportunities.

## INTRODUCTION

In all species, finite metabolic resources must be allocated toward three competing systems: survival (or maintenance), reproduction, and growth [1, 2]. All species incur a resource bias towards growth during development [2-4]. Growth is energetically costly, and diverts resources away from maintenance systems in favor of growth [5, 6]. However, even with energetically demanding growth periods, species should evolve processes that maintain physiological systems important to survival.

All birds grow rapidly [5, 6]. Short-lived species with low survival rates develop and reach sexual maturity quickly [6-8]. With a decreased annual survival rate; resource investment should favor growth and reproductive, rather than maintenance systems. In long-lived species, however, there is a fitness advantage to adult survival. While long-lived species will still incur the resource bias during growth, they should also allocate resources to systems that benefit increased longevity. Evidence of this tradeoff is known, as developmental conditions like brood size [9], growth rate [10] and being smaller than brood-mates [11] are shown to affect survival [12, 13]. We hypothesize that the continual investment cost in maintenance systems, like telomeres, increases the potential of adult survival and future reproductive events.

Telomeres are nucleoprotein complexes that protect the ends of chromosomes [14]. During each cycle of cell replication, telomeres are shortened because DNA polymerase cannot fully replicate the 3′ end of the DNA [14-16]. Telomere shortening is correlated negatively with adult survival in most species [17-20], and telomeres shorten more slowly in long-lived birds than in shorter-lived species [17]. In long-lived species, there is a fitness advantage to increased survival through more reproductive opportunities. Thus, long-lived species should allocate resources to increase adult survival, like telomeres.

In most species, growth is implicated in the shortening of telomeres. In King penguins (*Aptenodytes patagonicus*), chicks that had a faster growth rate showed higher oxidative damage and had accelerated telomere loss [10]. One explanation for the shortening is oxidative damage, which results in DNA damage and shorter telomeres [21-24]. Specifically, reactive oxygen species (ROS) produce DNA strand breaks, which are difficult to repair when they occur in telomeric DNA [24]. Reactive oxygen species are byproducts of mitochondrial metabolism, which is increased during growth [25, 26]. Thus, telomere shortening might be caused by the increase in mitochondrial metabolism during growth [24, 27]. While telomere length may shorten during the growth period, if telomere length and rate of shortening is important to survival, fledging with longer telomeres may maximize lifespan.

While telomeres shorten in most adult vertebrates, they can also be elongated by two primary mechanisms; (1) through the action of telomerase, a ribonucleic reverse transcriptase (TERT), or (2) homologous recombina-tion-mediated DNA replication, termed the Alternative Lengthening of Telomeres (ALT) [28, 29]. Telomerase contains both an enzyme with reverse transcriptase activity to elongate linear DNA as well as carries it's own RNA template [30-32]. Some vertebrates show increased telomerase activity during development [10, 31, 33, 34]. Additionally, increased telomerase activity in regions where cell lines develop could allow mature somatic cells to have longer telomeres as the individual ages. Telomeres in human sperm cells display this phenomenon. Sperm cell telomeres from older individuals are longer, as they are likely acted upon by enhanced telomerase activity in the gonads [35]. Additionally, telomerase activity is implicated in enhanced longevity observed in some taxa, including lobsters [36], *hydra* and *planaria* [37], of which the latter two are thought to be biologically immortal. A number of transcription factors regulate the activity of telomerase, so the expression is highly variable among cell conditions [38]. Oncogenes, however, can activate these transcription factors, and therefore telomerase is up-regulated in approximately 85-90% of cancer cases [38, 39].

Magellanic penguins (*Spheniscus magellanicus*) in the wild live more than 30 years [40], exceeding their maximum, mass-predicted lifespan by 26% [41]. We predict that, if growth is a stressor, and evolution favors adult penguins having the highest chance of survival, Magellanic penguins should finish growth with the longest possible telomeres. We quantified telomere length during growth of Magellanic penguins to characterize the impact of high cellular proliferation and energy demand on telomeres. We predicted that: (a) in young chicks, the lengths of telomeres would decrease but (b) telomere lengths would stop shortening by the time chicks reached fledging age.

## RESULTS

**Figure 1:**
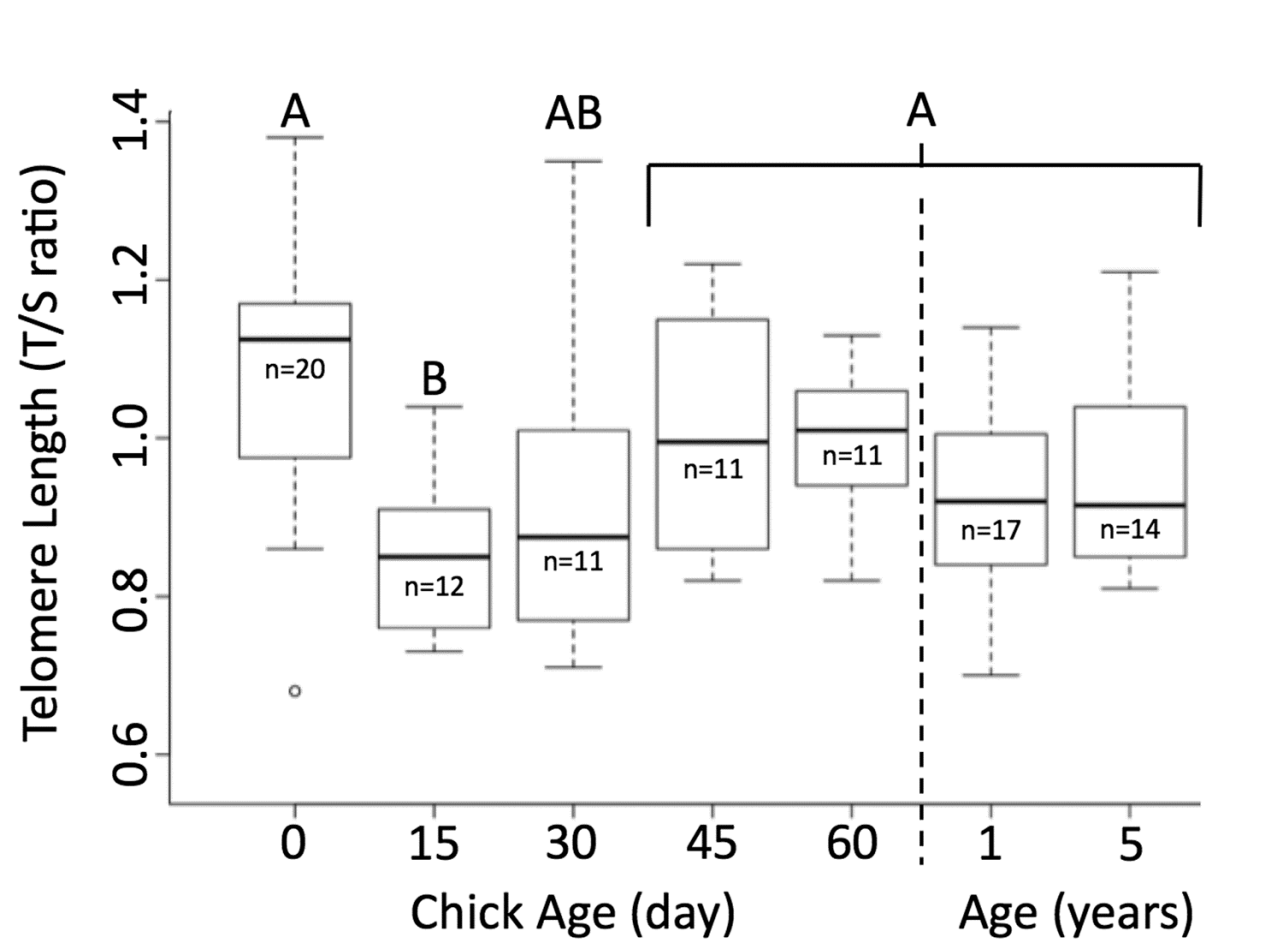
Telomere length shortens and then elongates in Magellanic penguins Telomere length shortens in early life, but returns to hatch day length by age 30 days. Groups (A/B) that do not share the same heading letter are significantly different. Dark bars within boxes represent group means, sample sizes presented under means, and bars are standard error.

Telomeres were significantly shorter when chicks were 15 days of age than when they hatched (Figure 1). By 30 days of age, telomere length was similar to their length at hatching, and they remained unchanged through 60 days of age (p=0.002, n=65, TukeyHSD, Figure 1). Telomeres of newly hatched chicks, 1-year-old juveniles, and adults aged 5 years were similar in length (p=0.14, n=51). There was no relationship between the change of bill depth, flipper length, foot length or weight and the change in telomere length between day 0 and day 15 (*BD*: Rsq = 0.246, p=0.07, n =11*; FL*: Rsq = 0.129, p = 0.15, n = 11*; FT*: Rsq = -0.076, p = 0.60, n = 11*; WT*: Rsq = 0.101, p = 0.18, n = 11). However, there was a significant correlation in the change of bill length and telomere length change from day 0 to day 15 (Rsq = 0.374, p = 0.026, n = 11). One chick, that had very little change between 0 and 15 days, drove this trend, so it is unlikely to be biologically meaningful. Those individuals that survived to fledging and those that died while nestlings had similar telomere lengths (t= 0.792, p=0.43, n=20).

## DISCUSSION

Telomeres shorten from hatching to day 15, but telomeres of newly hatched chicks, chicks ≥30 days, 1-year-old juveniles, and adults aged 5 years were similar in length. As was predicted, Magellanic penguin telomeres shorten immediately after hatch. Growth, in young chicks, requires high levels of energy and is characterized by high metabolism [5, 42, 43]. A high metabolic state can release reactive oxygen species (ROS), which shorten telomeres [24, 34]. This mechanism most likely explains what we observed between hatch day and day 15. King penguin chicks also showed a similar correlative link between growth and telomere shortening [10]. After day 15, however, Magellanic penguin telomeres elongated back to hatch day length by fledging and penguins entered adult size with telomeres of similar length to when they hatched.

This phenomena was not observed in King penguins [10]. King penguins live only 73% of their mass-predicted lifespan, where lifespan *= 17.6 (mass in kg)^0.20)* [41], shorter than Magellanic penguins [40, 44]. Since telomeres are linked to adult survival in most species, elongation of telomeres to hatch length by the time Magellanic penguins are adult size could contribute to this longevity [17-20]. In fact, we showed previously that Magellanic penguins conserve their telomere length from age 5 years to older than 27 years, even if they reproduce more (Cerchiara et al, in review).

Telomerase elongates telomeres [14, 45]. After birth in most mammals, telomeres in somatic cells begin shortening [46], and, in adults, telomerase is only found up-regulated in germ and stem cell lines and remains at low levels in somatic cell lines of kidneys, lymphocytes and epithelial cells, which is likely part of a tumor suppression mechanism [30, 32, 47, 48]. There is some evidence, however, that telomerase may remain active in some adult long-lived birds (*Sterna hirundo; Oceanodroma leucorhoa*) compared to short lived ones [45], which could explain the telomere elongation we observed as it is the only known reverse-transcriptase to elongate telomeres [30, 32, 47-53]. To our knowledge, late-growth elongation, like we observed, is only known for one species, the fish *Oryzias latipes* [54]. Increased telomerase activity is the proposed mechanism for developmental telomere elongation observed in *O. latipes* [54], so this is likely the same mechanism contributing to our results. Although we did not measure telomerase directly, we measured an increase in telomere length from shortest length on day 15 back to hatch day length by day 30, similar to what was shown in *O. latipes*.

Magellanic penguins elongated telomeres by the end of their growth period, making them one of the few species to evolve a physiological system that allows telomeres to recover to their original length at hatching. This mechanism allows Magellanic penguins to enter reproductive age with telomere lengths comparable to their length at hatching.

## MATERIALS AND METHODS

### Samples

In 1982, we began studying Magellanic penguins at Punta Tombo, Argentina (44°02′S, 65°11′W), banding chicks, juveniles, and breeding adults [55]. We know the lay date of individual eggs, and because we checked the nests daily before hatching, we know the exact hatch day of individual chicks [56].

We collected blood from wild Magellanic penguin chicks from hatching (November 2010) to fledging or death (January 2011). Chicks were marked on the day they hatched and bled on hatch day (day 0) and then captured and bled every 15 days until they were 60 days of age. Chick mortality reduced sample sizes during the nestling period: hatching day (“day 0”, n=20 chicks), 15 days old (n=12), 30 days old (n=11), 45 days old (n=11), and 60 days old (n=11). We also collected blood from 20 juveniles (1 year, January 2011). We collected blood from 15 known-age adult Magellanic penguins, aged 5 years from September to December 2007 at Punta Tombo, Argentina. We measured morphological traits (bill, flipper, foot and weight) of all individuals on the day of sample collection [55].

### Sample collection and processing

We collected whole blood into a heparinized capillary tube (Thermo Fisher Scientific Inc.), and immediately placed the blood into anti-lyses buffer (10% DMSO/90% Newborn Bovine Serum), and placed the samples on ice. We froze the samples at -18°C within 1hr of collection. We stored the samples at -80°C at the University of Washington, until processing.

We extracted DNA from a lightly centrifuged cell pack, consisting primarily of erythrocytes (Qiagen DNeasy Mini-kit), and then quantified DNA via nanodrop spectrophotometer (*mean 260/280 ratio±SE = 1.85±0.02)*. We measured telomere length by Quantitative Polymerase Chain Reaction (qPCR), a method used to measure telomere length in other penguins [10, 21].

### qPCR – telomere length

We ran two PCR assays for each sample. The first PCR amplified the telomeric DNA and the second amplified a single-copy control gene (36B4, acidic ribosomal phosphoprotein PO). The control gene PCR is used to normalize the starting amount of DNA. We included a four-point standard curve (2-fold serial dilutions from 10 to 1.25ng of DNA) in all PCRs to allow the transformation of Ct (cycle threshold) into nanograms of DNA. All samples were run in triplicates and for analyses we used the median value.

We ran all PCR reactions in a Rotor-Gene 3000 (Corbett Research, Sydney, Australia), with a final volume of 20ul including: 1X PCR buffer (Invitrogen, Carlsbad, CA), 0.2mM dNTPs, 0.4X SybrGreen (Molecular Probes, Eugene, OR), 2.5mM DTT, 1% DMSO and 5ng of DNA. The telomere PCR used 0.8ul of Platinum Taq (Invitrogen), 1.5mM MgCl, and 300nM of each primer (tel1b: CGGTTTGTTTGGGTTTGGGTTTGGGTTTGGGTTTGGTT; tel2b: GGCTTGCCTTACCCTTACCCTTAC CCTTACCCTTACCCT). PCR conditions were: 95°C for 15min and 30 cycles of amplification at 95°C for 15sec and at 56°C for 60sec. For the control gene, we used 36B4 primers designed with Primer3 with the 36B4 gene sequence in the common chicken (*Gallus gallus; NW_001471461.2)* and zebra finch (*Taeniopygia guttata; NW_002197395.1*) from the nucleotide database, GenBank. The control-gene PCR used 0.5ul of Platinum Taq, 3.5mM MgCl, 300nM of forward primer (MAPE1: AGGGAGAAGAGGGACTGGAC) and 500nM of reverse primer (MAPE2-CAATCCCACACACACCTCAG). Control gene PCR conditions were: 95°C for 15min and 35 cycles of amplification at 95°C for 15sec and at 56°C for 20sec and 72°C for 20sec. A melting (dissociation) curve, run at the end of every control-gene PCR, confirmed the presence of a single amplification product [57]. We verified the primers showed the same product (at ∼75bp) for all samples when ran on a 2% gel, with no secondary products. We targeted the 75bp oligomer of the 36B4 reference gene, so we are confident in the accuracy of our control gene [58].

We imported the raw data from the Rotor Gene software into Excel (v14.4.4) and aligned all amplification plots to a baseline height of 2% in the first 5 cycles of amplification. The fluorescence threshold for determination of the Ct was set at 20% of maximum products at the beginning of the exponential phase of the plot. We used a four point standard curve to calculate the corresponding nanograms for telomere analysis (mean telomere efficiency ± SE = 0.74±0.01, mean 36B4 efficiency ± SE = 0.88±0.02; all Rsq > 0.99). Due to lower efficiency values, we checked the absorbance curves for every sample and, if replicates were not overlapping (visual evaluation), we removed these data from our analyses. We re-ran removed samples again on additional trials, and evaluated the absorbance curves again.

The amount of telomeric DNA was divided by the amount of control-gene DNA, producing a relative measurement of the telomere length of the sample. For every trial, we ran two control samples for normalization between trials and to assess reproducibility of accurate measurements. The intra- and inter-trial variability (coefficient of variation) for the qPCR was 7% and 8%, respectively [59].

### Statistics

Previous work showed that, in 60 days, Magellanic penguins develop from newly hatched chicks (∼76g) to fledging weight (∼1800g) [55]. Since we used repeated samples of the same individuals during growth all analyses used a mixed-effects model approach with age (as a factor) as a fixed effect and chick-ID as a random effect to test if telomere length was predicted by chick age. We used a TukeyHSD to test for difference between age groups for chicks and for adults.

We used a linear model to determine if the growth rate (percent change in body part measurements between sample dates) of body parts predicted telomere length (percent change in telomere length). We also used a binomial linear model to determine in the telomere length at hatching predicted those chicks that fledged and those that died during growth. For statistical tests, we used R Statistical software (R Foundation for Statistical Computing: Development Core Team (v3.1.3).
